# Automated Arrhythmia Classification Using Farmland Fertility Algorithm with Hybrid Deep Learning Model on Internet of Things Environment

**DOI:** 10.3390/s23198265

**Published:** 2023-10-06

**Authors:** Ahmed S. Almasoud, Hanan Abdullah Mengash, Majdy M. Eltahir, Nabil Sharaf Almalki, Mrim M. Alnfiai, Ahmed S. Salama

**Affiliations:** 1 Department of Information Systems, College of Computer and Information Sciences, Prince Sultan University, Riyadh 12435, Saudi Arabia; 2 Department of Information Systems, College of Computer and Information Sciences, Princess Nourah bint Abdulrahman University, Riyadh 11671, Saudi Arabia; 3 Department of Information Systems, College of Science & Art at Mahayil, King Khalid University, Riyadh 12372, Saudi Arabia; meltahir@kku.edu.sa; 4 Department of Special Education, College of Education, King Saud University, Riyadh 12372, Saudi Arabia; 5 Department of Information Technology, College of Computers and Information Technology, Taif University, Taif 21944, Saudi Arabia; 6 Department of Electrical Engineering, Faculty of Engineering & Technology, Future University in Egypt, New Cairo 11845, Egypt; a.salama@fue.edu.eg

**Keywords:** Internet of Things, remote monitoring, arrhythmia classification, ECG signals, deep learning

## Abstract

In recent years, the rapid progress of Internet of Things (IoT) solutions has offered an immense opportunity for the collection and dissemination of health records in a central data platform. Electrocardiogram (ECG), a fast, easy, and non-invasive method, is generally employed in the evaluation of heart conditions that lead to heart ailments and the identification of heart diseases. The deployment of IoT devices for arrhythmia classification offers many benefits such as remote patient care, continuous monitoring, and early recognition of abnormal heart rhythms. However, it is challenging to diagnose and manually classify arrhythmia as the manual diagnosis of ECG signals is a time-consuming process. Therefore, the current article presents the automated arrhythmia classification using the Farmland Fertility Algorithm with Hybrid Deep Learning (AAC-FFAHDL) approach in the IoT platform. The proposed AAC-FFAHDL system exploits the hyperparameter-tuned DL model for ECG signal analysis, thereby diagnosing arrhythmia. In order to accomplish this, the AAC-FFAHDL technique initially performs data pre-processing to scale the input signals into a uniform format. Further, the AAC-FFAHDL technique uses the HDL approach for detection and classification of arrhythmia. In order to improve the classification and detection performance of the HDL approach, the AAC-FFAHDL technique involves an FFA-based hyperparameter tuning process. The proposed AAC-FFAHDL approach was validated through simulation using the benchmark ECG database. The comparative experimental analysis outcomes confirmed that the AAC-FFAHDL system achieves promising performance compared with other models under different evaluation measures.

## 1. Introduction

The Internet of Things (IoT) involves intercommunication among a wide variety of smart devices such as sensor devices, laptops, smartphones, and personal digital assistants (PDAs) [1]. Amongst different domains of IoT applications, healthcare is one of the major domains that has gained attention in recent years. The wide possibilities of the IoT have resulted in the development of various medical applications to assist with chronic diseases, older people care, fitness programs, and remote health monitoring [2]. However, another important and possible application of IoT in the healthcare domain is conformity with medication and therapy at home by medical professionals. In this background, diagnostic devices, imaging devices, sensors, and medical equipment are considered smart devices and form the IoT platform [3]. IoT-based medical facilities have the potential to reduce the costs incurred upon diagnosis and improve the user skills and quality of life. In the aspect of medical staff, the IoT can utilize remote requirements so as to reduce the device’s downtime. Electrocardiograph (ECG) is the Cartesian representation of the electrical signals produced by the heart. ECG can be employed in measuring the heartbeat’s rate and regularity, and the chamber’s size and position [4]. This simple non-invasive technique can be exploited to detect a few heart diseases (HD) and analyze the impact of drugs or heart regulatory devices. ECG devices with changing counts of electrodes (three to twelve) are utilized for signal acquisition, while the ECG signals are nonstationary in nature. Due to this characteristic, a medical specialist or a cardiologist might fail to examine the heart’s condition [5]. According to the WHO, Cardiovascular Diseases (CVDs) are the main cause of death around the world. Among CVD-related deaths, cardiac arrhythmia is the main contributor. Disturbances that occur in the heart rate, due to abnormal impulse formation or electrical conduction in the heart, are named under ‘arrhythmia’.

IoT devices have been developed and utilized in a number of applications for improving, communicating, and monitoring purposes [6]. Moreover, they provide data at the exact time without discussion with medical experts, and this phenomenon might be highly effective in rural regions [7]. Arrhythmia cases are detected by gathering signals from individuals and measuring them with the help of an analytical instrument, an ECG. Some of the significant processes conducted for the classification of arrhythmia include feature selection (FS), feature extraction, and classification [8]. The problem to overcome is that the classification process must be executed even with unbalanced data. Various optimization techniques are employed in the optimization of the hyperparameters of a classifier. The Deep Neural Network (DNN) enables the extraction of higher-level features, which are required for the identification of arrhythmia without human interpolation [9]. Wearable medical devices have been developed to reduce the mortality rates that occur due to HD arrhythmia by measuring blood pressure (BP) level and by monitoring the heartbeat, body temperature, and other such physical health factors [10].

The current research article presents the automated arrhythmia classification using a Farmland Fertility Algorithm with Hybrid Deep Learning (AAC-FFAHDL) approach in the IoT platform. The presented AAC-FFAHDL technique exploits the hyperparameter-tuned DL model for ECG signal analysis, thereby detecting the arrhythmia. To accomplish this, the AAC-FFAHDL technique initially performs data pre-processing to scale the input signals into a uniform format. Further, the AAC-FFAHDL technique uses HDL for the detection and classification of arrhythmia. In order to improve the classification and detection results of the HDL algorithm, the AAC-FFAHDL technique involves an FFA-based hyperparameter tuning process. The AAC-FFAHDL system was experimentally validated using the benchmark ECG database. In short, the major contributions of the current research work are listed herewith.

- Presentation of an automated AAC-FFAHDL technique comprising pre-processing, HDL-based classification, and FFA-based hyperparameter tuning for arrhythmia classification. To the best of the authors’ knowledge, the AAC-FFAHDL model has never been presented in the literature.
- Employment of the HDL model for the classification process, which leverages the benefits of both CNN and GRU models.
- Hyperparameter optimization of the HDL model using the FFA algorithm through a cross-validation method helps in boosting the predictive outcomes of the AAC-FFAHDL model for unseen data.

## 2. Related Works

Kumar et al. [11] proposed an efficient classification method for the classification of HD by employing the flamingo optimization technique. Primarily, the ECG signal from the heart was gathered, after which it was exposed to the pre-processing phase for detecting and controlling the electrical signal of the heart. The input signals were gathered by exploiting the IoT nodes, which can altogether exist in BS for classification by employing flamingo-optimizer-based DCNs. In the study conducted earlier [12], an automatic classification model was proposed for the identification of arrhythmia using the optimized DL-classifier. In this study, the ECG signals gathered utilizing the IoT nodes were processed to produce the difficult QRS and RR intervals to determine the feature vectors. The latter task was performed using the developed Coy–Grey Wolf optimizer-based deep CNN (Coy–GWO-based Deep CNN) classifier for the detection of anomalies in ECG signal. Jagadeesh et al. [13] introduced an automatic arrhythmia classification technique by applying a Harris Hawks optimizer-based DL (AC-HHODL) approach in the IoT. In this study, the MobileNetv2 algorithm was implemented to generate a group of feature vectors. Further, the HHO approach was employed for optimal hyperparameter modification of the MobileNetv2 algorithm. In addition to these, the least square-SVM (LS-SVM) method was also exploited for accurate classification of arrhythmia.

Karthiga and Abirami [14] presented an ECG surveillance system by employing the IoT using two phases. Primarily, a routing protocol was developed based on Routing by Energy and Link quality (REL) and Dynamic Source Routing (DSR) for IoT medical platforms. Secondarily, the classification of the ECG signal was executed. This study compared the proposed method with other methods such as ANN, CNNs, and SVM-based techniques for classifying the ECG signals. Alnaggar et al. [15] introduced a hybrid design combining two models, namely, the ECG Heartbeat Multiclass Classification model (ECG-HMCM) and the Heart Attack Detection Model (HADM). In this study, Gridsearch was employed for the optimization of the hyperparameters for various ML approaches. In this architecture, the HADM database was deployed while the KNN algorithm was employed to make both ECG-HMCM as well as the Gridsearch method applicable for hyperparameter optimization. In literature [16], a new technique was introduced for the identification of the SVCA using the ECG signals. The Fixed Frequency Range Empirical Wavelet Transform (EWT) (FFREWT) filter bank was proposed for multi-scale examination of the ECG signals. The framework of the developed deep CNN had a total of four dense layers, four convolution layers, and two pooling layers. 

Kumar et al. [17] developed a structure that allows the emergence of real diagnostic instruments to detect cardiac arrhythmia in actual time. In this study, the ECG signal was processed utilizing the Pan Tompkins QRS (Quantum Resonance System) identification technique for the extraction of the dynamic features of the signal. In the study conducted earlier [18], an IoT system was developed to monitor the ECG signals and process the heart data. Firstly, the IoT surveillance systems and communication among the parts were determined. Secondly, the study method trained and tested three classification approaches, which are the KNNs, RF, and the CNN. 

Hemalatha [19] designed a new AdaBoost kernel-SVM-based remora optimization (AKSVM-based RO) technique for analyzing the ECG signals and initializing the crisis with the least possible time delay. The algorithm developed in this study was able to estimate the ECG signals of an arrhythmia-impacted individual from the IoT wearable device. In literature [20], a new cloud-based arrhythmia identification utilizing the Recurrent Neural Network (RNN) (NC-RNN) approach was developed for conducting the ECG analysis using wearable sensors in a smart city background. The ECG signal, gathered from the wearable sensor, was made to undergo a three-stage diagnostic phase. In the study conducted earlier [21], an integrated 1D-CNN technique was introduced. The interpretation component of the developed method can adapt itself through the fog structure so as to identify the ECG signals and establish emergency actions within the shortest time. However, its training method can be implemented only on the computationally improved cloud data centers. Lu et al. [22] presented the LSTM technique for increasing the accuracy of the diagnostic procedure. A classification technique was developed in this study for arrhythmia based on the CNN-LSTM framework. Primarily, the deep-CNN model was developed for encoding the ECG signals and extracting the morphological features of the ECG signals. Secondarily, the necessary features were deeply mined utilizing the temporal correlative LSTM approach by learning the morphological representation of the features. In literature [23], the authors extracted the non-linear and linear features gathered from the 5G-assisted medical IoT devices to make a time–frequency spectrogram from HRV sequences. This study employed a DL method based on the incorporation of a deep-CNN and an LSTM method for the classification of ARR intervals and normal sinus intervals. Yadav et al. [24] designed an innovative technique to monitor and analyze cardiac arrhythmia by employing the IoT platform. The authors also exploited a novel Fuzzy Logic (FL)-based-NN classifier method for the identification of cardiac arrhythmia.

## 3. The Proposed Model

**Figure 1:**
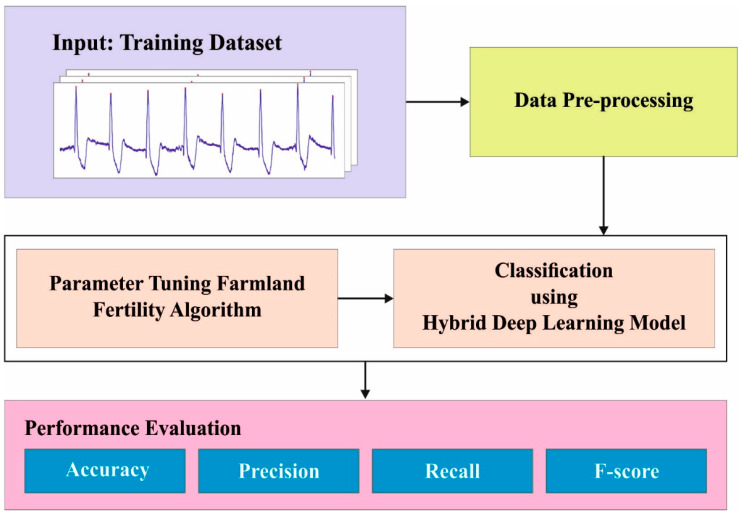
Overall procedure of the AAC-FFAHDL methodology.

In the current research work, a novel AAC-FFAHDL approach is presented for automated arrhythmia classification in the IoT platform. The presented AAC-FFAHDL system exploits the hyperparameter-tuned DL model for ECG signal analysis, thereby detecting arrhythmia. To accomplish this, the AAC-FFAHDL technique follows a three-step procedure, namely, data pre-processing, HDL-based classification, and FFA-based hyperparameter tuning. Figure 1 depicts the entire procedure of the proposed AAC-FFAHDL method.

### 3.1. Data Pre-Processing

In this stage, the input ECG data are employed to detect arrhythmia in its early stages. The data are pre-processed following a typical scalar transform approach so that normalized data are achieved and the unrelated noisy data are eliminated. Various approaches exist for data normalization such as averaging, min–max scaling, and standard scaling. In the current study, a typical scalar is employed for normalizing the data. The standard scalar employs the Standard Normal Distribution (SND), where its mean is 0 and variance is 1. The mathematical model is defined in Equation (1).

(1) $$z=\frac{x-\mu }{sd}\;\;\;$$

where *sd* implies the Standard Deviation, *z* denotes the typical feature space of the *x* input data instances, and *μ* denotes the mean.

### 3.2. Arrhythmia Detection Using HDL Model

In this stage, the HDL approach is executed for the diagnosis and classification of arrhythmia. The HDL approach contains CNN-GRU methods. The upgraded LSTM structure is termed as GRU. To regulate data flow, the GRUs also take a gate infrastructure similar to LSTM. However, unlike the LSTMs, GRU does not have an output gate; thus, it allows the content to be completely revealed. The update and reset gates can obtain only two gates from the GRU. The forget gate and the input gate of the LSTM infrastructure can be integrated into a second gate. With regard to LSTM, GRUs take a simple infrastructure and any parameter, if introduced, enhances the outcome. 

The formulas below provide the GRU equation.

(2) $$r_{t}=sigm\left(W_{xr}x_{t}+W_{hr}h_{t-1}+b_{r}\right)$$

(3) $$z_{t}=sigm\left(W_{xz}x_{t}+W_{hz}h_{t-1}+b_{z}\right)\;$$

(4) $${\tilde{h}}_{t}=tanh\left(W_{xh}x_{t}+W_{hh}\left(r_{t}⊙h_{t-1}\right)+b_{h}\right)\;$$

(5) $$h_{t}=z_{t}⊙h_{t-1}+\left(1-z_{t}\right)⊙{\tilde{h}}_{t})$$

where ‘$h_{t}$’, ‘$x_{t}$’, ‘$z_{t}$’, and ‘$r_{t}’$ denote the output and input vectors, and the update and reset gates, respectively. Like LSTM, ‘b’ implies the bias; ‘W’ defines the weighted layer; and ‘sigm’ and ‘tanh’ represent the sigmoid and tangent functions, respectively. Either LSTM or GRU controls the longer dependencies. In this case, it can be utilized for both structures to evaluate its potential to classify the network traffic.

The fundamental CNN model contains three major modules: the convolution, pooling, and output layers. Amongst these, the pooling layer can be chosen. A typical CNN infrastructure contains three convolutional layers and is extensively employed in image classification tasks. It takes one input layer, many Hidden Layers (HLs) (HLs contain pooling, normalization, and convolutional), and an FC layer to the final layer named as the ‘resultant layer’. The response to the next layer is computed with the support of mathematical convolutional functions of the 1st or input layer. 

**Figure 2:**
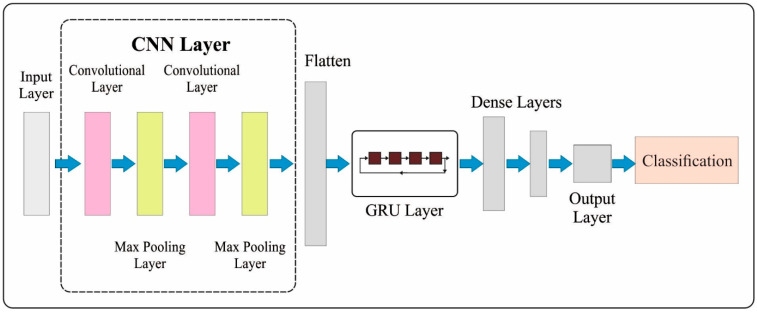
CNN-GRU structure.

The CNN-GRU approach contains three convolutional layers (C) along with two GRU layers (G) and one HL (H). It employs 32 neurons for the C layer and 64 neurons for the G layer. The neuron for the final layer (resultant layer) corresponds to the labels from the database. G and C layers employ the ReLU function for activation. Figure 2 displays the framework of CNN-GRU. The softmax $\left(S_{-}\max\right)$ activation function, which offers the outcome with respect to prediction probability, is employed in the last layer. With the deployment of the softmax layer, the vector of the numbers is changed as a vector of probabilities; however, all the probability values are inversely proportional to its comparative scale. 

### 3.3. Parameter Tuning using FFA

In order to optimally adjust the parameters of the HDL approach, the FFA technique is applied. FFA is a recent metaheuristic approach inspired from the natural fertility of agricultural land. In the FFA technique, the solution gets enhanced with the division of agricultural land. The solution of every section is optimized using the optimum exploitation of the internal and external memory. Firstly, the primary population is produced in a random manner and a primary parameter is set. Unlike the other approaches, the solution is also divided. Next, the count of initial population is described as follows:

(6) N=k×n 

Here, k shows the amount of land space or parts and  n specifies the overall amount of solution in the search space. At this stage, the solution available in the search space is assessed by FF. The FFA technique takes a separate part to define the k value that shows the optimum value for 8 ≥k≥2. However, according to the optimization problem, the k value gets changed. At first, the solution is allocated to various parts using the following equation:

(7) $$Section_{s}=x\left(aj\right),\;a=n\left(s-1\right):\;n\times s,\;$$

$$s=\left\{1,2,\;\ldots k\right\},\;j=\left\{1,2,\;\ldots D\right\}$$

Here, the present solution separates every part to determine the average of every section. s indicates the section number, x denotes the solution in the search space, and j={1,2, … D} refers to the dimension of the variable x. The quality of every part is defined by Equation (3) after segmentation.

(8) $$Fit_{-}Section_{s}=Mean\left(allfit\;\left(x_{ji}\right)in\;Section_{s}\right)$$

$$s=\left\{1.2\ldots \ldots k\right\}.\;i=\left\{1.2\ldots \ldots n\right\}$$

Here, $Fit_{-}Section$ shows the value that defines the solution’s quality in every section of the agricultural land that is similar to the average fit or suitability of the solution. Thus, for every agricultural land part, the average solution within every part is attained and stored in $Fit_{-}Section_{s}.$ Equation (9) is used for updating the local memory and Equation (10) is exploited for global memory.

(9) $$M_{Global}=round\left(t\times N\right);\;0.1<t<1\;\;$$

(10) $$M_{local}=round\left(t\times n\right);\;0.1<t<1$$

where $M_{Global}$ and $M_{local}$ denote the solution counts from global and local memory solutions, respectively, based on suitability and fitness. Furthermore, these two memories are also updated. The section with the worst quality variations show most of the work progressions. The worst part of agricultural land in terms of quality is integrated with a global memory solution based on the following expression:

(11) $$h=\alpha .rand\left(-1.1\right)\;$$

(12) $$Xnew=h.\left(X_{\mathrm{ij}}-X_{MGlobal}\right)+X_{\mathrm{ij}}$$

where $X_{MGloba1}$ refers to random solutions available in global memory and α denotes the number within [0.1]. $X_{i_{\dot{j}}}$ shows the performance in the worst part of the agricultural lands chosen to execute the alters, and h indicates the decimal value evaluated using Equation (11). Based on Equations (13) and (14), the solutions in other sections tend to change.

(13) $$h=\beta .rand\left(0.1\right)\;\;$$

(14) $$Xnew=h\times \left(X_{i_{\dot{j}}}-X_{U\dot{j}}\right)+X_{i_{\dot{j}}}$$

Here, $X_{U\dot{j}}$ denotes the arbitrary solution amongst the available solutions in the searching space and β refers to a number in the range of [0,1]. $X_{ij}$ indicates the solution from the part chosen to execute the alters, and h represents a decimal number evaluated using Equation (14). The hybridization of the desired resolution with $Best_{Global}$ or $Best_{Local}$ is defined as follows:

(15) $$H=\left\{\begin{array}{l} X_{new}=X_{ij}+\omega_{1}\left(X_{ij}-Best_{Global}\left(b\right)\right)if\;Q>rand\\ X_{new}=X_{ij}+rand\left(0.1\right)\times \left(X_{ij}-Best_{Local}\left(b\right)\right)\;else \end{array}\right.\;$$

Here, Q refers to a parameter initialized within [0,1]. This parameter shows the extent to which the solution is combined $t_{Global}.$ $\omega_{1}$.

(16) $$\omega_{1}=\omega_{1}.R_{v},0<R_{v}<1\;$$

According to the objective function, the solution available throughout the search space is assessed. Notwithstanding the number of sections, this step is executed on the solution available in the search space. The terminal condition is checked at the end. If the ending criteria is reached, the process ends; otherwise, the process is continued until the terminal condition is reached.

The FFA methodology develops the Fitness Function (FF) for realizing the best classifier solution. It explains a positive integer to signify a good solution for candidate performances. In this effort, the minimized classifier rate of the errors is supposed to be the FF, as written in Equation (17).

(17) $$fitness\left(x_{i}\right)=ClassifierErrorRate\left(x_{i}\right)=\frac{No.\;of\;misclassified\;instances\;}{Total\;no.\;of\;instances}*100$$

## 4. Results and Discussion

The proposed AAC-FFAHD algorithm was validated through simulation using the MIT-BIH dataset (https://physionet.org/content/mitdb/1.0.0/, accessed on 14 June 2023). The proposed model was simulated using Python 3.6.5 on a PC equipped with an i5-8600k, GeForce 1050Ti 4 GB, 16 GB RAM, 250 GB SSD, and 1 TB HDD. The parameter settings are as follows: learning rate, 0.01; dropout, 0.5; batch size, 5; epoch count, 50; and activation, ReLU. For experimental validation, different training/testing dataset ratios were used: 40:60, 50:50, 60:40, 70:30, and 80:20.

**Figure 3:**
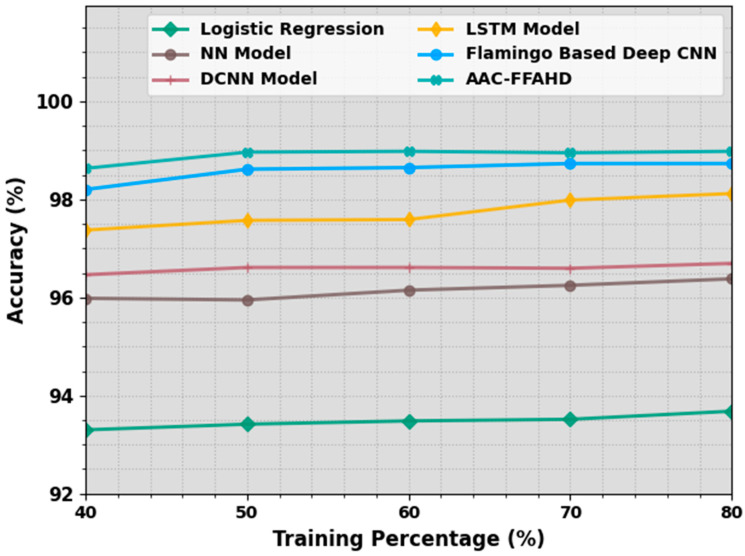
$Accu_{y}$ outcomes of the AAC-FFAHD system on varying TR values.

In Table 1 and Figure 3, the comparative $accu_{y}$ investigation outcomes of the AAC-FFAHD system are shown [11]. The simulation values highlight that the proposed AAC-FFAHD method reached better outcomes with varying training (TR) values. With 40% TR data, the AAC-FFAHD technique obtained a maximum $accu_{y}$ of 98.63%, whereas the LR, NN, DCNN, LSTM, and F-DCNN models attained the least $accu_{y}$ values of 93.30%, 95.98%, 96.46%, 97.37%, and 98.20%, respectively. In addition, with 50% TR data, the AAC-FFAHD approach accomplished a superior $accu_{y}$ of 98.96%, whereas the LR, NN, DCNN, LSTM, and F-DCNN methods attained the lowest $accu_{y}$ values of 93.42%, 95.95%, 96.61%, 97.57%, and 98.62%, respectively. Afterwards, with 80% TR data, the AAC-FFAHD system yielded an improved $accu_{y}$ of 98.98%, whereas the LR, NN, DCNN, LSTM, and F-DCNN algorithms achieved low $accu_{y}$ values of 93.68%, 96.38%, 96.70%, 98.12%, and 98.73%, respectively.

**Figure 4:**
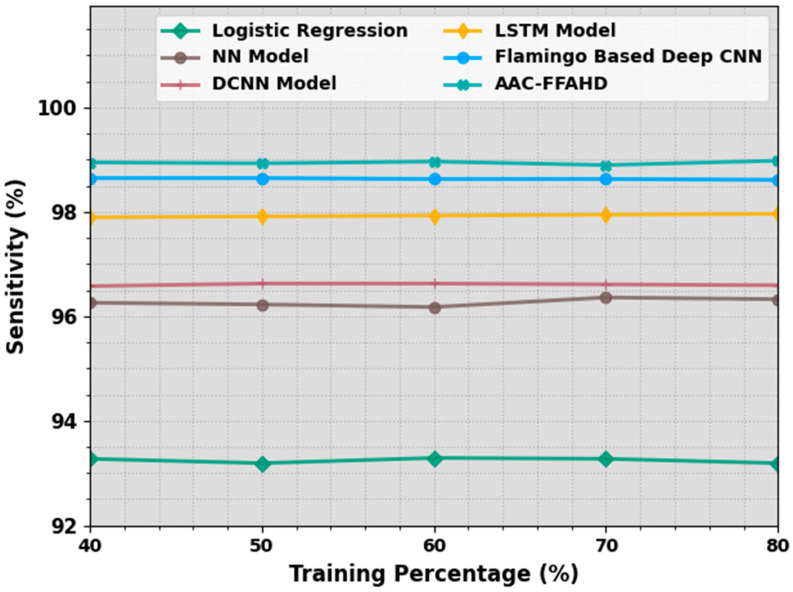
$Sens_{y}$ outcomes of the AAC-FFAHD system on varying TR values.

In Table 2 and Figure 4, the detailed $sens_{y}$ investigation outcomes achieved by the proposed AAC-FFAHD algorithm are shown. The simulation values define that the AAC-FFAHD system reached a better performance with varying TR values. With 40% TR data, the AAC-FFAHD approach gained a superior $sens_{y}$ of 98.95%, whereas the LR, NN, DCNN, LSTM, and F-DCNN models achieved minimal $sens_{y}$ values of 93.27%, 96.26%, 96.58%, 97.90%, and 98.65%, respectively. Also, with 50% TR data, the AAC-FFAHD technique obtained a higher $sens_{y}$ of 98.93%, whereas the LR, NN, DCNN, LSTM, and F-DCNN models attained low $sens_{y}$ values of 93.19%, 96.23%, 96.63%, 97.91%, and 98.65%, respectively. Eventually, with 80% TR data, the AAC-FFAHD system yielded a maximum $sens_{y}$ of 98.98%, whereas the LR, NN, DCNN, LSTM, and F-DCNN approaches reached the minimal $sens_{y}$ values of 93.19%, 96.33%, 96.59%, 97.96%, and 98.61%, respectively.

**Figure 5:**
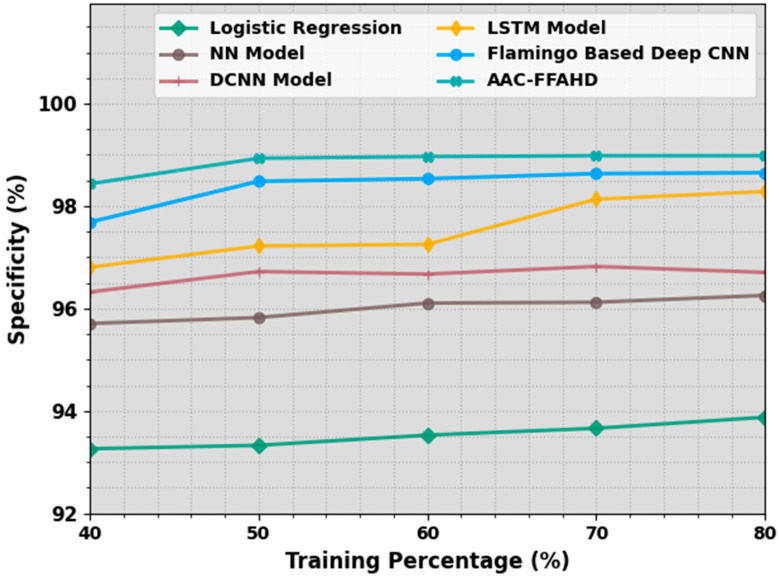
$Spec_{y}$ outcomes of the AAC-FFAHD system on varying TR values.

Table 3 and Figure 5 portray the comparative $spec_{y}$ analysis results achieved by the proposed AAC-FFAHD algorithm and other techniques. The simulation values demonstrate that the AAC-FFAHD system reached the optimum results with varying TR values. With 40% TR data, the AAC-FFAHD technique obtained a superior $spec_{y}$ of 98.43% whereas the LR, NN, DCNN, LSTM, and F-DCNN systems gained low $spec_{y}$ values of 93.26%, 95.71%, 96.32%, 96.80%, and 97.69%, respectively. Additionally, with 50% TR data, the AAC-FFAHD system obtained an improved $spec_{y}$ of 98.93%, whereas the LR, NN, DCNN, LSTM, and F-DCNN approaches reached the minimal $spec_{y}$ values of 93.33%, 95.82%, 96.72%, 97.22%, and 98.48%, respectively. Next, with 80% TR data, the AAC-FFAHD method achieved an enhanced $spec_{y}$ of 98.98%, whereas the LR, NN, DCNN, LSTM, and F-DCNN approaches accomplished low $spec_{y}$ values of 93.88%, 96.26%, 96.70%, 98.28%, and 98.65%, respectively.

**Figure 6:**
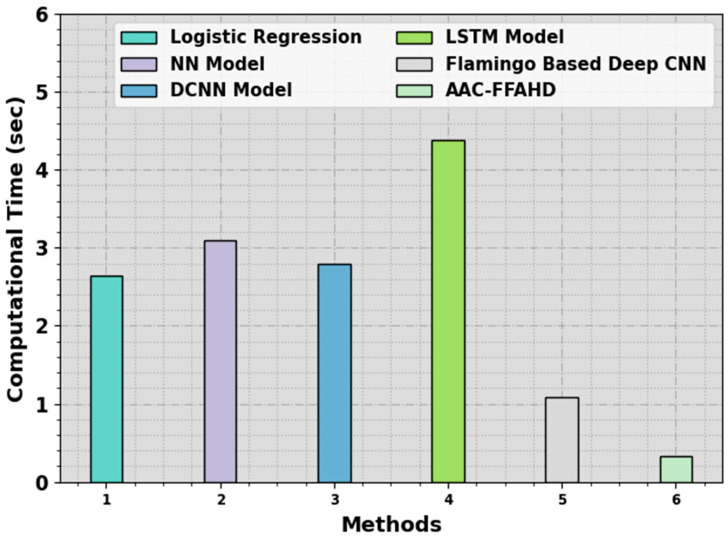
CT outcomes of the AAC-FFAHD system and other methods.

Table 4 and Figure 6 show the comparative analytical outcomes of the AAC-FFAHD system and other algorithms in terms of Computation Time (CT). The simulation values imply that the AAC-FFAHD method took the least CT of 0.33s. At the same time, the LR, NN, DCNN, LSTM, and flamingo-based Deep CNN models presented long CT values of 2.64 s, 3.09 s, 2.80 s, 4.38 s, and 1.08 s, respectively. Thus, it can be inferred that the AAC-FFAHD technique can be employed for accurate and automated classification. 

**Figure 7:**
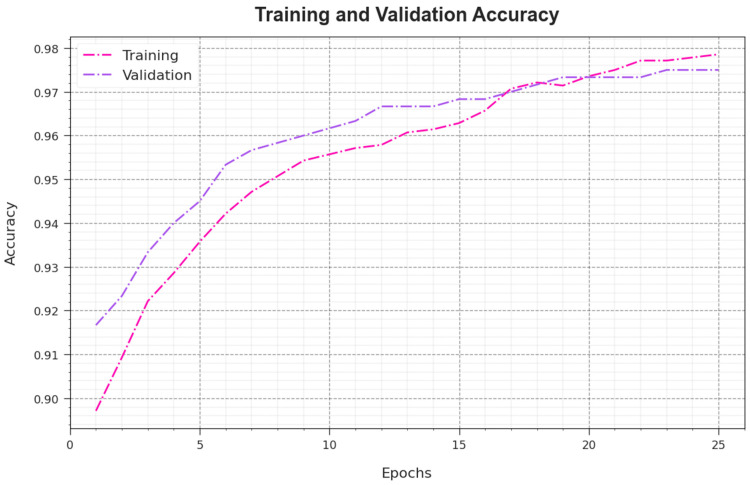
$Accu_{y}$ curve of the AAC-FFAHD system.

Figure 7 demonstrates the training accuracy $TR\_accu_{y}$ and $VL\_accu_{y}$ values of the AAC-FFAHD methodology. The $TL\_accu_{y}$ is measured by evaluating the AAC-FFAHD technique on the TR dataset, whereas the $VL\_accu_{y}$ is computed by estimating the solution using a separate testing dataset. The outcomes depict that both $TR\_accu_{y}$ and $VL\_accu_{y}$ increase with an increase in the number of epochs. Accordingly, the performance of the AAC-FFAHD method was superior on the TR and TS datasets with an increase in the count of epochs.

**Figure 8:**
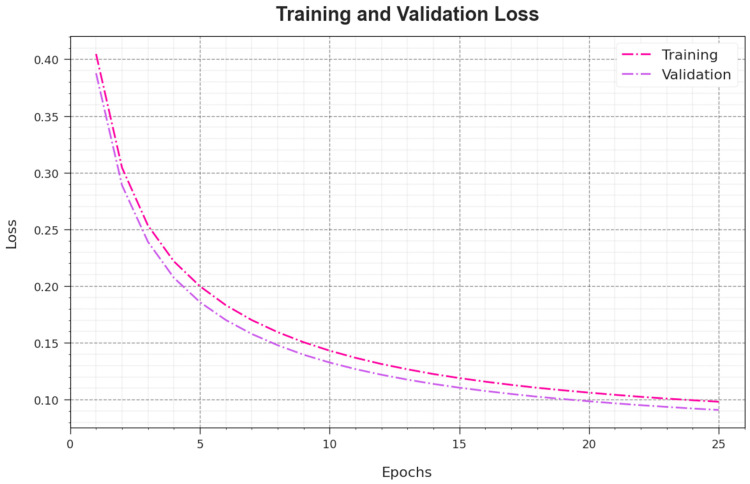
Loss curve of the AAC-FFAHD system.

In Figure 8, the TR_loss and VR_loss curves of the AAC-FFAHD algorithm are shown. The TR_loss demonstrates the error between the predictive outcome and original values of the TR data. The VR_loss signifies the performance of the AAC-FFAHD technique on individual validation data. The outcomes show that the TR_loss and VR_loss values decrease with an increasing number of epochs. This outcome depicts the enhanced performance of the AAC-FFAHD technique and its ability to make accurate classifications. The low TR_loss and VR_loss values demonstrate the supreme performance of the AAC-FFAHD technique in terms of capturing the patterns and relationships.

**Figure 9:**
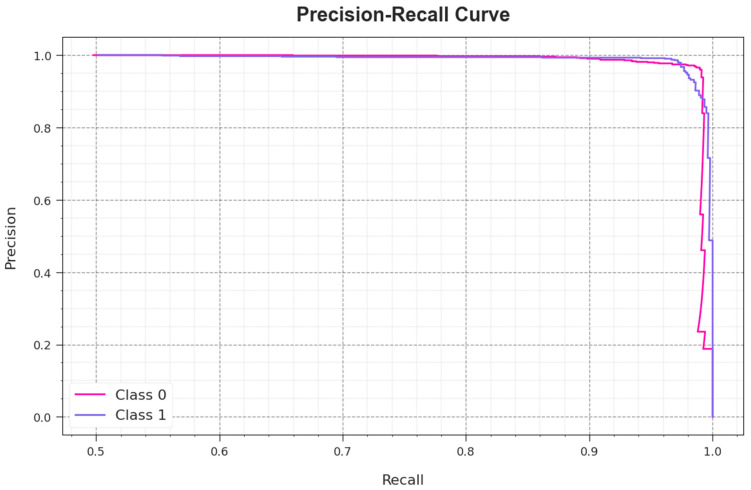
PR curve of the AAC-FFAHD system.

A comprehensive Precision–Recall (PR) examination was conducted upon the AAC-FFAHD approach using the test database, and the results are shown in Figure 9. The simulation values infer the superior performance of the AAC-FFAHD algorithm in enhancing the PR values. Thus, it is clear that the AAC-FFAHD approach can attain superior PR values in two class labels.

**Figure 10:**
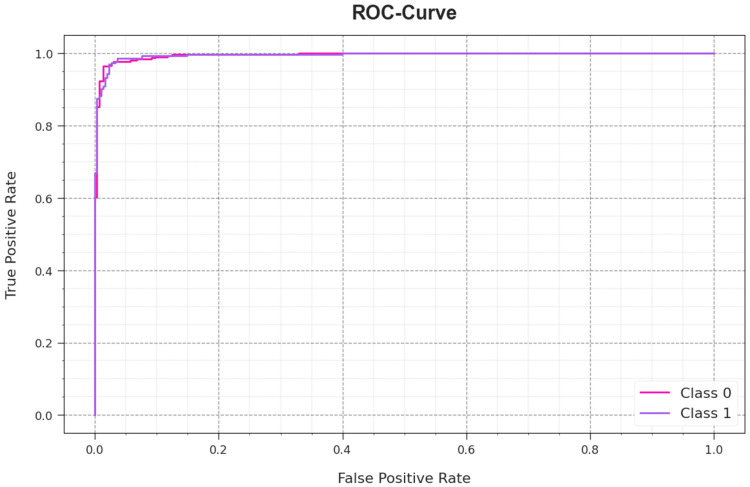
ROC curve of the AAC-FFAHD system.

In Figure 10, an ROC investigation curve of the AAC-FFAHD algorithm using the test database is demonstrated. The simulation value depicts that the AAC-FFAHD methodology achieved higher ROC values. Moreover, it is apparent that the AAC-FFAHD method achieved high ROC values in two classes.

**Figure 11:**
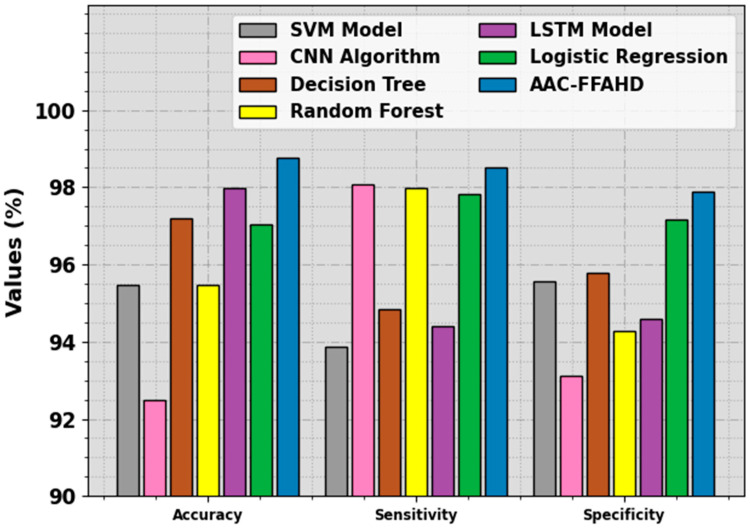
Comparative results of the AAC-FFAHD technique and other techniques on the CU dataset.

In order to further validate the performance of the proposed model, a detailed analysis was conducted on CU: The Creighton University Sustained Ventricular Arrhythmia Database [25]. Table 5 and Figure 11 offer the comparative analysis outcomes achieved by the proposed model and other recent approaches on the CU dataset. The figure indicates that the RF, SVM, and CNN models obtained the worst performance over other models. At the same time, the DT, LSTM, and LR models attained slightly improved results. However, the AAC-FFAHD model accomplished superior performance with a maximum $accu_{y}$ of 98.76%, $sens_{y}$ of 98.50%, and $spec_{y}$ of 97.90%. Therefore, the AAC-FFAHD technique can be applied for automated and accurate classification of arrhythmia.

In summary, the simulation outcomes of the AAC-FFAHD technique demonstrate accurate and automated classification of arrhythmia. The enhanced performance of the AAC-FFAHD model is due to the incorporation of the HDL technique and FFA-optimizer-based hyperparameter tuning. The FFA chooses the optimal values for the hyperparameters of a given HDL model. Hyperparameters are settings that are not learned during the training process and must be set prior to training. This scenario has a significant impact on the performance of the model. Further, the selection of optimal values can lead to better accuracy. By leveraging the FFA-based hyperparameter tuning process, the AAC-FFAHD model achieved better results by focusing on the most relevant features and selecting the optimal settings for the algorithm. These results establish the improved performance of the AAC-FFAHD technique over other existing techniques.

## 5. Conclusions

In the current study, a novel AAC-FFAHDL methodology is presented for automated arrhythmia classification in the IoT environment. The presented AAC-FFAHDL approach exploits the hyperparameter-tuned DL model for ECG signal analysis, thereby detecting arrhythmia. To accomplish this, the AAC-FFAHDL approach follows a procedure containing three steps: data pre-processing, HDL-based classification, and FFA-based hyperparameter tuning. In order to enhance the classification and detection performance of the HDL approach, the AAC-FFAHDL technique involves the FFA-based hyperparameter tuning process. The AAC-FFAHDL system was experimentally validated through simulation using the benchmark ECG database. The comparative experimental analysis outcomes confirm that the AAC-FFAHDL method accomplished more promising performance than the rest of the models in terms of various evaluation measures. In the future, the performance of the AAC-FFAHDL technique can be enhanced by FS methods. Since healthcare data are sensitive, it is essential to have robust data privacy and security measures within the IoT platform. Future works should focus on addressing these concerns and complying with relevant regulations. Moreover, adaptive learning techniques can be designed that allow the model to learn and adapt over time as it encounters new data and the scenarios can further improve its performance and reliability. Enhancing the interpretability of the model’s predictions is crucial, especially in healthcare applications. So, future work must focus on developing novel methods to explain the model’s decisions, providing clinicians with insights about the classification process of a particular arrhythmia. 

## Figures and Tables

**Table 1:** $Accu_{y}$ outcomes of the AAC-FFAHD system and other methods [11] on varying TR values.

Accuracy (%)						
Training Percentage (%)	Logistic Regression	NN Model	DCNN Model	LSTM Model	Flamingo-Based Deep CNN	AAC-FFAHD
40	93.30	95.98	96.46	97.37	98.20	98.63
50	93.42	95.95	96.61	97.57	98.62	98.96
60	93.48	96.15	96.61	97.59	98.65	98.98
70	93.52	96.25	96.60	97.99	98.73	98.95
80	93.68	96.38	96.70	98.12	98.73	98.98

**Table 2:** $Sens_{y}$ outcomes of the AAC-FFAHD system and other methods on varying TR values.

Sensitivity (%)						
Training Percentage (%)	Logistic Regression	NN Model	DCNN Model	LSTM Model	Flamingo-Based Deep CNN	AAC-FFAHD
40	93.27	96.26	96.58	97.90	98.65	98.95
50	93.19	96.23	96.63	97.91	98.65	98.93
60	93.29	96.18	96.63	97.93	98.63	98.96
70	93.27	96.36	96.61	97.95	98.63	98.90
80	93.19	96.33	96.59	97.96	98.61	98.98

**Table 3:** $Spec_{y}$ outcomes of the AAC-FFAHD system and other methods on varying TR values.

Specificity (%)						
Training Percentage (%)	Logistic Regression	NN Model	DCNN Model	LSTM Model	Flamingo-Based Deep CNN	AAC-FFAHD
40	93.26	95.71	96.32	96.80	97.69	98.43
50	93.33	95.82	96.72	97.22	98.48	98.93
60	93.53	96.11	96.67	97.25	98.53	98.97
70	93.66	96.12	96.82	98.13	98.63	98.98
80	93.88	96.26	96.70	98.28	98.65	98.98

**Table 4:** CT outcomes of the AAC-FFAHD technique and other methodologies.

Methods	Computational Time (sec)
Logistic Regression	2.64
NN Model	3.09
DCNN Model	2.80
LSTM Model	4.38
Flamingo-Based Deep CNN	1.08
AAC-FFAHD	0.33

**Table 5:** Comparative results of the AAC-FFAHD technique and other models on the CU dataset.

Method	Accuracy	Sensitivity	Specificity
SVM Model	95.46	93.87	95.56
CNN Algorithm	92.50	98.09	93.13
Decision Tree	97.19	94.83	95.78
Random Forest	95.46	97.99	94.27
LSTM Model	97.99	94.40	94.60
Logistic Regression	97.03	97.83	97.17
AAC-FFAHD	98.76	98.50	97.90

## Data Availability

Data sharing is not applicable to this article as no datasets were generated during the current study.
